# New insights into the treatment of real *N*,*N*-dimethylacetamide contaminated wastewater using a membrane bioreactor and its membrane fouling implications

**DOI:** 10.1039/c8ra01657g

**Published:** 2018-04-04

**Authors:** Maoshui Zhuo, Olusegun K. Abass, Kaisong Zhang

**Affiliations:** CAS Key Laboratory of Urban Pollutant Conversion, Institute of Urban Environment, Chinese Academy of Sciences 1799 Jimei Road Xiamen 361021 China mszhuo@iue.ac.cn +86-13159256518; University of Chinese Academy of Sciences Beijing 100049 China

## Abstract

Treatment of *N*,*N*-dimethylacetamide (DMAC) wastewater is an important step in achieving the sustainable industrial application of DMAC as an organic solvent. This is the first time that treatment of a high concentration of DMAC in real wastewater has been assessed using membrane bioreactor technology. In this study, an anoxic–oxic membrane bioreactor (MBR) was operated over a month to mineralize concentrated DMAC wastewater. Severe membrane fouling occurred during the short-term operation of the MBR as the membrane flux decreased from 11.52 to 5.28 L (m^2^ h)^−1^. The membrane fouling was aggravated by the increased amount of protein fractions present in the MBR mixed liquor. Moreover, results from the excitation–emission matrix analysis identified tryptophan and other protein-like related substances as the major membrane-fouling components. Furthermore, analysis of the DMAC degradation mechanism *via* high performance liquid chromatography (HPLC) and ion chromatography (IC) revealed that the major degradation products were ammonium and dimethylamine (DMA). Although the MBR system achieved the steady removal of DMAC and chemical oxygen demand (COD) by up to 98% and 80%, respectively at DMAC_0_ ≤ 7548 mg L^−1^, DMA was found to have accumulated in the treated effluent. Our investigation provides insight into the prospect and challenges of using MBR systems for DMAC wastewater degradation.

## Abstract 2

Treatment of *N*,*N*-dimethylacetamide (DMAC) wastewater is an important step in achieving the sustainable industrial application of DMAC as an organic solvent.

## Graphical abstract

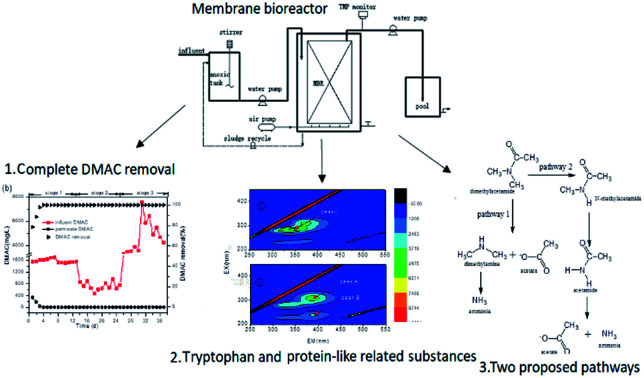

## 1. Introduction

*N*,*N*-Dimethylacetamide (DMAC) is a strong polar aprotic solvent that is completely miscible with water, ether, acetone, ester and so on, has high thermal stability, is difficult to hydrolyze, and is toxic. DMAC is widely applied in the manufacture of coatings, fibers, foils, lacquers and film fabrication.^1,2^ Some surveys have revealed that human DMAC exposure could lead to liver damage, skin irritation, headache, loss of appetite, and fatigue.^3,4^ Consequently, discharge of DMAC-containing wastewater can cause serious environmental pollution and harm to human health due to its typically high concentration in water, even after DMAC recovery.^5^ With the current revolution in the membrane industry, DMAC wastewater discharge and reclamation will soon become the next hot research topic.

Concentrations of DMAC in wastewater of as high as 20 000 mg L^−1^ are characteristic of the discharge water from large-scale polymeric membrane factories. Interestingly, until now, only a few research works have focused on the treatment of DMAC wastewater. Also, DMAC recovery is an effective energy-saving alternative and the waste DMAC could be converted into energetic materials, as carbon derived from DMAC waste has already been utilized in power generation for the production of 100 MW L^−1^ power density in microbial fuel cells at a potential of 0.45 V.^6^ Moreover, the DMAC removal efficiency was between 15% and 50% with a hydraulic retention time (HRT) of 12 min. An internal microelectrolysis process has been applied to treat DMAC wastewater at an influent concentration of 50 mg L^−1^, resulting in a DMAC removal rate of 95%.^7^ Nevertheless, technologies for DMAC removal at higher concentrations are currently lacking.

As a pure culture, the isolated *Rhodococcus* sp. strain B83 has been confirmed to biodegrade DMAC without the need for any extra source of carbon and nitrogen, reaching a degradation efficiency of 96.1% in 120 hours when the initial DMAC concentration was 15 000 mg L^−1^.^5^ However, at a DMAC concentration of greater than 15 000 mg L^−1^, a significant adverse effect on the pH of the culture was observed, which was harmful to the growth of the *Rhodococcus* sp. strain B83.^5^ Hence, it is indispensable to assess alternative technologies able to reclaim wastewater containing high levels of DMAC.

Membrane bioreactors (MBRs) are thought to be suitable for the treatment of DMAC wastewater due to their advantages over conventional candidates, which include the ability to process higher biomass concentrations, a smaller carbon footprint, less sludge generation, and better membrane permeability.^8–10^ Membrane bioreactors are generally used for both municipal and industrial wastewater treatment.^11,12^ For instance, a laboratory-scale submerged anoxic–oxic membrane bioreactor has been operated continuously to treat simulated wastewater contaminated with DDA (dianilinodithiophosphoric acid), an organic toxic flotation reagent, and the chemical oxygen demand (COD) removal efficiency rose up to 80% only after the system reached stability within a HRT of 4 h.^13^ The most challenging issue in the application of MBRs is the widely known problem of fouling.^14^ Research on membrane fouling during the treatment of wastewater with high concentrations of pollutants has been well documented.^15,16^

In this work, the potential of MBR has been exploited for the treatment of DMAC-containing wastewater. The study set out to achieve the following goals: (1) to assess the performance of the anoxic–oxic-MBR over a range of DMAC concentrations, (2) to explore the effects of DMAC loading rates on membrane fouling, and (3) to study the degradation mechanism of DMAC based on the identification of catabolic intermediates. To the best of our knowledge, this is the first time that DMAC removal by MBR has been assessed, while the fouling implications remain largely unknown. Results from this study will be beneficial for the further development of a MBR process for the treatment of DMAC polluted wastewater.

## 2. Experimental set-up

### 2.1 Raw wastewater

DMAC containing wastewater was collected from a membrane manufacturing company (Oxiamembrane Co., Ltd) in Xiamen, Fujian province, China. The wastewater was stored in large volume tanks at room temperature. Table 1 lists some characteristics of the DMAC raw wastewater.

**Table:** Some characteristics of the raw DMAC-containing wastewater^a^

Characteristics	Measurements
pH	5.4 ± 0.05
COD (mg L^−1^)	18 924 ± 2553
BOD_5_ (mg L^−1^)	1930
DMAC (mg L^−1^)	9910 ± 684
TOC (mg L^−1^)	5375 ± 249
TN (mg L^−1^)	2067 ± 96
NH_4_^+^–N (mg L^−1^)	<0.08
Li (mg L^−1^)	23.9 ± 0.5
Conductivity (mg L^−1^)	466.5 ± 3.5
Turbidity (NTU)	0.694

a COD: Chemical oxygen demand, BOD_5_: biochemical oxygen demand for 5 days, TOC: total organic carbon, TN: total nitrogen.

### 2.2 Inoculum

The inoculum used in the experiments was taken from Jimei wastewater treatment plant in Xiamen. The sludge was acclimatized with an appropriate amount of glucose, ammonium chloride, monopotassium phosphate (KH_2_PO_4_) and 500 mg L^−1^ of DMAC, in a batch state for 10 days. The influent wastewater (polluted with DMAC) was diluted and mixed well with KH_2_PO_4_ in a ratio of 200 : 1 to ensure the growth of microorganisms. After the addition of the phosphorus source, the pH of the wastewater in the tank was around 7.

### 2.3 Anoxic–oxic membrane bioreactor (A/O-MBR) set-up and operation

**Fig. 1:**
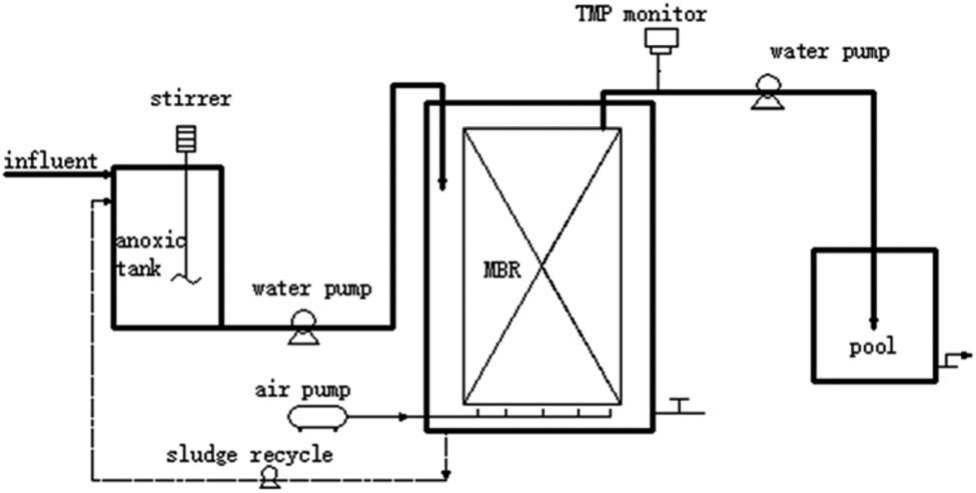
A process flow diagram of the DMAC wastewater treatment.

Fig. 1 shows the A/O-MBR process flow diagram operated on a semi-pilot scale. The effective volume of the anoxic tank and MBR are 28 L and 55 L, respectively. The MBR operation was divided into three stages, according to different input DMAC concentrations. The transmembrane pressure (TMP) was monitored online and controlled to be below 25 kPa. Samples of 1 L per day were taken prior to sludge discharge. More detailed set-up parameters and operating conditions are shown in Table 2.

Experimental parameters and operating conditions^a^

**Table:** 

Parameter	Stage 1	Stage 2	Stage 3
Time (days)	1–13	14–25	26–37
HRT_MBR_ (h)	24	24	24
HRT_Anoxic_ (h)	0	10	10
SRT (d)	—	55	55
Recycle ratio	—	200%	200%
DMAC (mg L^−1^)	1500–1700	800	3000
MLSS_MBR_ (mg L^−1^)	8558 ± 433	11 897.5 ± 922.5	15 898.8 ± 303.8
MLVSS_MBR_ (mg L^−1^)	5421 ± 24	7913.8 ± 1171.3	12 496.3 ± 98.8
MLSS_Anoxic_ (mg L^−1^)	—	8353.87 ± 46.3	8610
MLVSS_Anoxic_ (mg L^−1^)	—	5606.3 ± 856.3	6627.5
pH	8.3 ± 0.3	7.2 ± 0.3	8.6 ± 0.3
DO_MBR_ (mg L^−1^)	0.24–5.64	0.24–6.14	0.25–0.32
DO_Anoxic_ (mg L^−1^)	—	≤0.5	≤0.5
Air flow rate (L min^−1^)	15	15	15

a HRT: Hydraulic retention time, SRT: sludge retention time, MLSS: mixed liquid suspended solids, MLVSS: mixed liquid volatile suspended solids, DO: dissolved oxygen.

**Table:** 

Suction time	Run: 10 min, pause: 2 min
Membrane process	Micro-filtration, flat-sheet membrane plates
Membrane area (m^2^)	0.25
Membrane pore size (μm)	0.35
Membrane material	Polyvinylidene fluoride (PVDF)

### 2.4 Analytical methods

Chemical analysis including COD, total organic carbon (TOC) and total nitrogen was conducted using colorimetric COD test kits (Lianhua Tech. Co., Ltd., China)^17^ and a TOC-V_CPH_ analyzer (Shimadzu, Japan), respectively. The DMAC concentration was determined by high performance liquid chromatography (HPLC) equipped with a UV-vis diode array detector and extended-C18 column (5 μm, 150 × 4.6 mm) in reverse phase mode. The HPLC separation was executed within 7 min using CH_3_COONH_4_ (0.02 mol L^−1^) : CH_3_OH = 90 : 10 for an injection amount of 20 μL at a flow rate of 1.0 mL min^−1^, with an absorption wavelength of 200 nm and a column temperature of 40 °C.^1^ The amount of ammonium was measured by ion chromatography (Agilent 7890A, ICS). The amounts of mixed liquid suspended solid (MLSS) and volatile suspended solid (MLVSS) were analyzed by following a standard method.^18^

Sampling from the MBR bulk sludge was performed once every three days. As for extracellular polymeric substances (EPS) on the membrane surfaces, the fouling layer materials were carefully scraped off from two different areas of membrane surfaces at the end of stages 1 and 3 and were then dissolved in 40 mL of demineralized water for subsequent EPS extraction procedures. Extraction of the soluble microbial product (SMP) and EPS was conducted using a modified thermal method.^19^ 40 mL of activated sludge taken from the mixed liquid and membrane surface was first centrifuged at 6000*g* for 10 min. The supernatant was considered as the SMP. The remaining procedures of EPS extraction were conducted.^20^ The polysaccharide (PS) and protein (PT) content in both the SMP and EPS were measured using a sulfuric acid anthrone colorimetric method^21^ and BCA Protein Assay Kit,^22^ respectively. Besides this, the SMP and EPS were characterized using excitation–emission matrix (EEM) fluorescence spectroscopy. Different peaks in the EEM appeared at corresponding intersections of the excitation–emission wavelengths depending on the different types of functional groups present. A 3D scan fluorescence spectrophotometer (F-4600, HITACHI) with a PMT voltage of 650 V was applied for measuring the EEM spectra. The excitation (Ex) and emission (Em) sampling interval was 3.0 nm with a slit of 5.0 nm.

Identification of the DMAC degradation products was conducted using of ion chromatography (Agilent 7890A ICS and Dionex ICS-3000) and HPLC. All of the *N*,*N*-dimethylformamide (DMF), dimethylamine (DMA), *N*-methylacetamide (MMAC), acetamide, acetaldehyde and acetate standards were prepared in advance to verify the existence of acetate, dimethylamine, *N*-methylacetamide, and acetamide. Samples prepared using real wastewater contaminated with *N*,*N*-dimethylacetamide were tested to exclude instrument interference in the DMAC degradation.

## 3. Results and discussion

### 3.1 Treatment performance of the semi-pilot A/O-MBR set-up

**Fig. 2:**
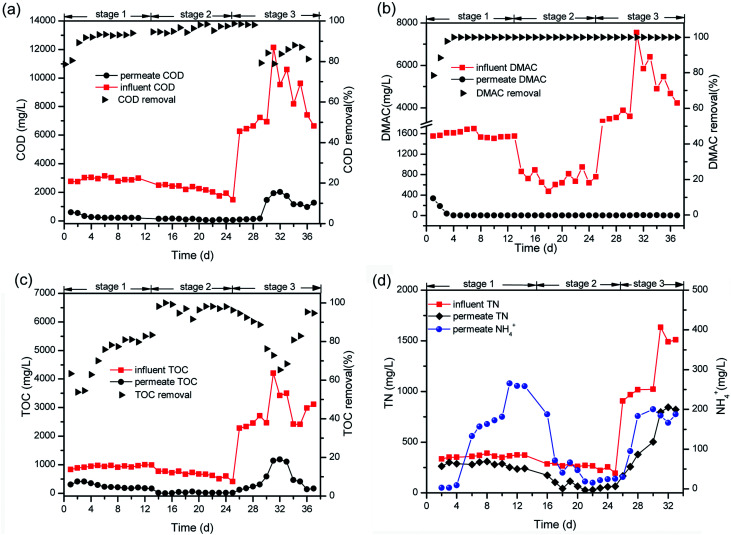
Variation of treatment indicators in the MBR effluent including (a) COD, (b) DMAC, (c) TOC, and (d) NH_4_^+^, and the TN removal efficiencies.

Treatment of the DMAC wastewater was conducted in three stages. Firstly, the MBR was singly operated for 13 days to explore the treatment performance and mechanism of the DMAC degradation in an aerobic environment. At the initial stage, the DMAC, COD and TOC removal efficiencies increased steadily reflecting the gradual stability of the MBR system, as shown in Fig. 2. After a short-term acclimatization, stable removal values for the COD, TOC and DMAC of 92.85 ± 1.15%, 76.7 ± 6.7% and 100% were achieved, respectively, at DMAC concentrations ranging from 1500 to 1700 mg L^−1^. A dosage of 800 mg L^−1^ of DMAC was applied in stage 2 and the DMAC loading rate was decreased to assess the removal characteristics of the MBR at a low concentration of DMAC. Furthermore, ahead of stage 2, pre-acclimatization of the anoxic sludge was conducted for ten days (data not shown) prior to coupling with the MBR. This was done to enhance Total Nitrogen (TN) removal, as a high concentration of ammonium was detected in the effluent. The removal of DMAC (100%) was steadily maintained while COD (96.15 ± 2.25%) and TOC (95.5 ± 4.5%) removal was greatly improved at a low DMAC concentration (Fig. 2a–c). Moreover, the ammonium concentration in the effluent decreased relative to the DMAC influent concentration (Fig. 2d). As previously reported, degradation of DMAC by the *Rhodococcus* strain B83 produces ammonia as one of its by-products.^5^ Therefore, to assess the influence of the coupled anoxic reactor on the ammonia and nitrogen removal characteristics, the concentration of the DMAC influent was increased in the next treatment phase.

At the third stage, the DMAC removal efficiency still reached 100% regardless of the increase in the influent concentration (from 800 to 3346 mg L^−1^ and then continuously to 7548 mg L^−1^). Similar characteristic removal of a toxic constituent by MBR has been previously reported,^23,24^ where elevated concentrations of antibiotics were consistently removed and the shock loading effect of DMAC did not affect the removal performance.^25^ The TN removal rate also improved relative to the influent DMAC concentration (Fig. 2d). Interestingly, regardless of the variation in the influent DMAC concentration, its removal by the MBR system remains constant. Hence, our results demonstrates that treatment of DMAC wastewater using a MBR system could withstand the influence of influent fluctuation, which is a common characteristic of various industrial discharges, despite the low biodegradability index (BOD_5_/COD = 0.1) of the raw wastewater.

### 3.2 Membrane fouling in the DMAC-fed A/O-MBR

**Fig. 3:**
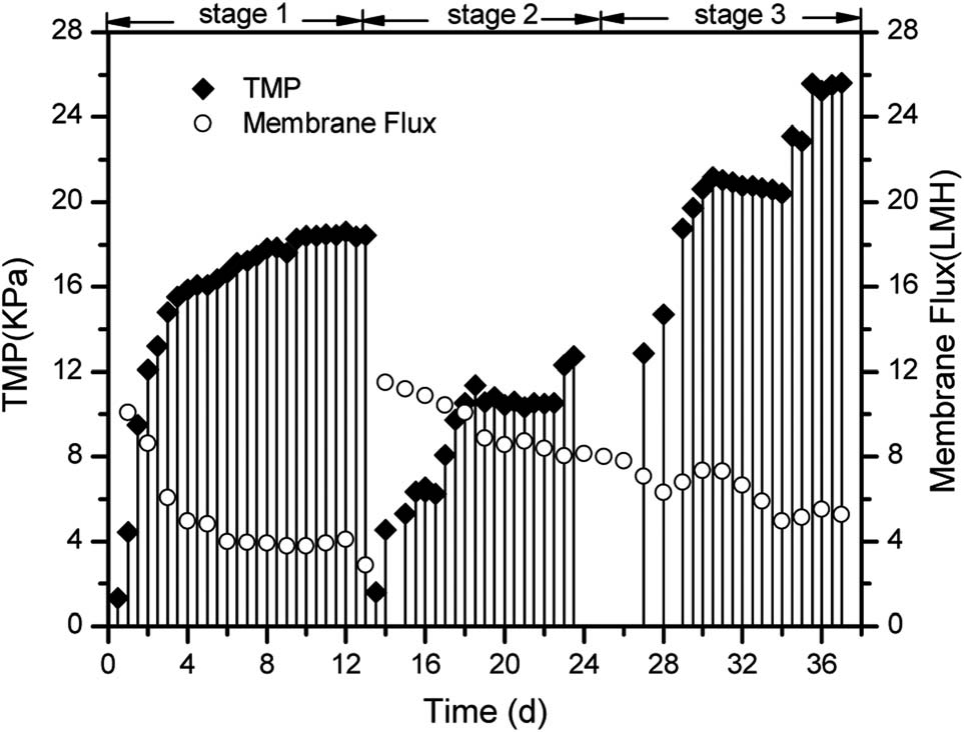
Transmembrane pressure and membrane flux profile.

**Fig. 4:**
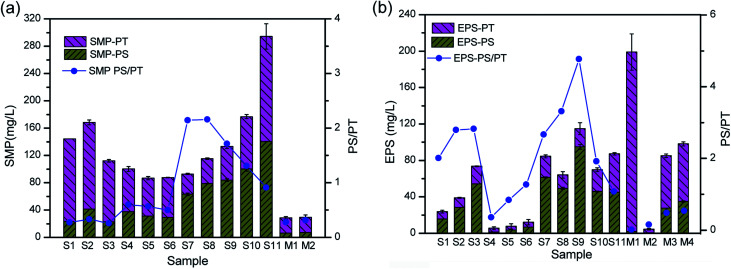
Concentration of polysaccharides and proteins and their ratios at different treatment stages. (a) The SMP and (b) EPS, where samples S1, S2, and S3 represent stage 1, S4, S5, and S6 represent stage 2, S7 S8, S9, S10, and S11 represent stage 3, samples M1 and M2 represent the membrane surface at the end of stage 1 and samples M3 and M4 represent the membrane surface at the end of stage 3.

Membrane fouling is affected by four factors: the membrane materials, biomass characteristics, feedwater characteristics, and operating conditions.^26–28^ In this study, we focused on the different interactions between the biomass products and membrane surface. Severe membrane fouling occurred during short-term A/O-MBR operation, as shown in Fig. 3. During the MBR start-up operation, the TMP rose from 1 kPa to 9 kPa with a corresponding decrease in the flux during the first stage of the operation. This is not very common in most MBR operations and is less common than the observations made by previous researchers.^27,29^ A probe into the chief cause of the rapid fouling of the membrane revealed that the bulk MBR sludge contains a high concentration of the SMP protein fraction (126 ± 3.57 mg L^−1^), as shown in Fig. 4a. This early fouling event has however been associated with high filtration resistance of the gelation layer produced by relatively high SMP concentration.^30^

The initial high SMP concentration in the MBR bulk sludge is mainly due to the influent DMAC concentration (1500 mg L^−1^). A variety of microbial products are formed due to changes in the microbial activity in non-steady state conditions and the microorganisms will generally secrete SMP and EPS to protect their fragile membrane from damage in stress conditions.^31,32^ As described in Section 1, the microorganisms completely adapt to the new environment after three days. However, the TMP steadily increased, while the flux varied inversely with the TMP. In a similar study, it was reported that exposure of microorganisms to pharmaceutical compounds increased the production of SMPs.^33^ At stage 2, the membrane flux was recovered by physical cleaning and was re-inserted into the MBR set-up. In contrast, the TMP values were lower at stage 2 owing to a decrease in the influent DMAC concentration (800 mg L^−1^). The potential toxicity of DMAC has been well studied.^4,34^ Hence, it is speculated that the lower fouling rate corresponds to the low SMP and EPS produced during this stage (Fig. 4).

At stage 3, a sudden rise in the TMP and subsequent membrane flux reduction occurred after the influent DMAC increased to 3000 mg L^−1^, as shown in Fig. 3. Similarly, the membrane flux decreased from 11.52 to 5.28 LMH. Thus, effective membrane fouling control measures are paramount for MBR operation in constant flux mode when treating wastewater contaminated with high levels of DMAC. For example, mechanical cleaning using fluidized particles, such as beads and biofilm carriers, can be practically applied with potential, particularly for flat-sheet membrane modules.^14^ In the same vein, anti-fouling membrane materials could be considered.^35^ Similarly, addition of a pre-treatment unit such as hydrolysis and advanced oxidation treatment, could help to increase the biodegradability of DMAC contaminated wastewater. As such, soluble foulants released by microorganisms in response to DMAC toxicity will be reduced, thus mitigating membrane fouling. In recent work by J. K. Xue *et al.*, it was shown that using ozone for the pretreatment of oil sands process-affected water prior to MBR treatment effectively mitigated membrane fouling.^36^

### 3.3 SMP, EPS and EEM analysis

EPS in either a bound or soluble form plays a predominant role in membrane fouling in MBRs.^27^ SMP and EPS as fouling-causing substances in MBRs containing numerous molecules or compounds (*e.g.* polysaccharides and proteins), which are potentially involved in inter-molecule or inter-component interactions.^14^ Characterization of membrane fouling in this study was conducted by quantitatively analyzing the polysaccharides and proteins fractions in the SMP and EPS. As shown in Fig. 4, the variation in the EPS concentration was consistent with DMAC loading. EPS production by live bacteria under stressed conditions could easily aid their attachment potential onto membrane surfaces compared to that of dead bacteria.^37^ Therefore, the increase in the bound EPS concentration has been directly related to an increase in the specific cake resistance, which consequently leads to a rise in the TMP.^38,39^

The SMP PS/PT ratio in the bulk sludge rose from 0.6 to 2, and finally decreased down to 0.9 at stage 3. Likewise, the EPS PS/PT ratio increased from 0.4 to 4.7 and then decreased down to 1.0. It was found that the SMP and EPS PS/PT ratios were lower than 1.0 when severe membrane fouling occurred during the treatment of raw oil sands process-affected water (OSPW) without pretreatment.^36^ The SMP and EPS PS/PT ratios on the fouled membrane surface were lower than 0.55, as shown in Fig. 4, which indicates that protein fractions contribute more to the fouling layer. Fouled membranes are dominated by biopolymers, including SMP-polysaccharides and EPS-proteins, in the early and late stages of fouling.^14^ Therefore, as shown in this work, protein fractions played a key role in the fouling of the membrane.

**Fig. 5:**
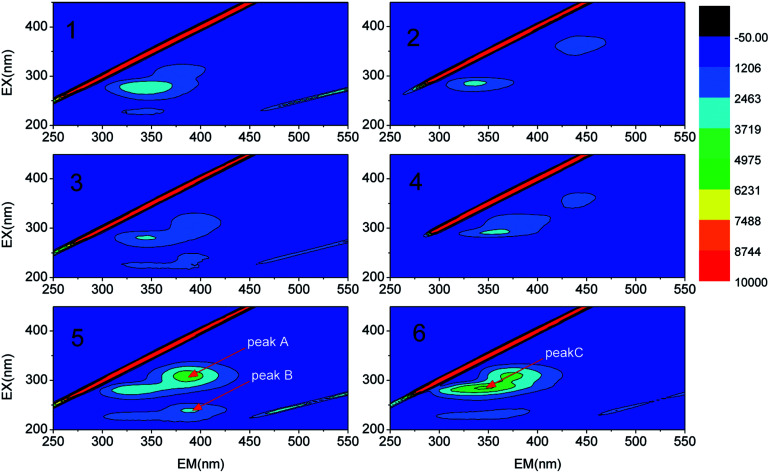
EEM fluorescence spectra of the SMP (1 and 2) and EPS (3 and 4) in mixed-liquor suspended sludge and the EPS on the membrane surface (5 and 6).

To demonstrate the role of inter-foulant species (*e.g.* polysaccharides, proteins and humic substances) on membrane fouling during the MBR operation, the EEM fluorescence spectra of the SMP and EPS were acquired (Fig. 5). A high intensity of peak C at an Ex/Em wavelength of 287/350 was found to be dominant among the three main peaks and has been identified in the literature as being due to the presence of tryptophan and other protein-like related substances.^40–43^ Peak A and peak B, located at Ex/Em wavelengths of 311/385 and 239/388, have been previously ascribed to being due to the presence of marine humic and fulvic acid-like species.^33,42^ These two peaks intermittently appeared during the analysis of the SMP and EPS compositions. Current research^44,45^ is focused on a potential approach for achieving effective fouling control through the selection and cultivation of polysaccharide-degrading bacteria or enzymes. Thus, bioaugmentation of the MBR with specific bacteria or enzymes capable of degrading the dominant protein-like foulant responsible for peak C will be an effective strategy for mitigating membrane fouling in DMAC treatment using a MBR.

To evaluate the membrane rejection efficiency of the polysaccharides and proteins in the MBR system, concentrations of the PS and PT in the sludge supernatant and permeate were measured and are presented in Table 3. Complete retention of the PT fractions was observed at stages 1 and 2, and only small amounts of the PS and PT fractions were detected in the permeate. Biomass commonly utilizes organic material along with the generation of SMPs in biological treatment reactors.^46^ In another report, it was observed that SMPs contribute to the majority of the unremoved COD in effluent wastewaters.^46^ However, few studies have shown the possibility of SMP retention by MBR.^47,48^ Thus, MBR is a promising technology for the treatment of DMAC contaminated wastewater, although membrane fouling is still a common shortcoming to be overcome.

**Table:** Concentration and membrane rejection efficiency of the polysaccharides and proteins in the suspended liquor and effluent

Samples	PS_MBR_ (mg L^−1^)	PS_permeate_ (mg L^−1^)	PT_MBR_ (mg L^−1^)	PT_permeate_ (mg L^−1^)
S1	23.54	0.00	120.62	0.00
S2	41.71	0.00	126.57 ± 3.57	0.00
S3	22.91	0.00	89.11 ± 2.00	0.00
S4	37.56	2.28	62.68 ± 3.57	0.00
S5	31.21	0.00	55.40 ± 2.38	0.00
S6	29.12	6.81	58.39 ± 0.55	0.00
S7	63.27 ± 1.76	4.98 ± 0.39	29.51 ± 0.93	0.00
S8	78.82 ± 0.27	6.34 ± 0.63	36.50 ± 1.138	2.03 ± 0.34
S9	84.00 ± 1.87	7.22 ± 0.13	49.03 ± 2.91	2.76 ± 0.21
S10	100.20 ± 3.27	6.16 ± 0.57	76.56 ± 2.98	1.95 ± 0.41
S11	140.34 ± 0.19	4.91 ± 0.26	153.97 ± 18.49	2.59 ± 0.08

### 3.4 Mechanistic pathways of DMAC degradation

**Fig. 6:**
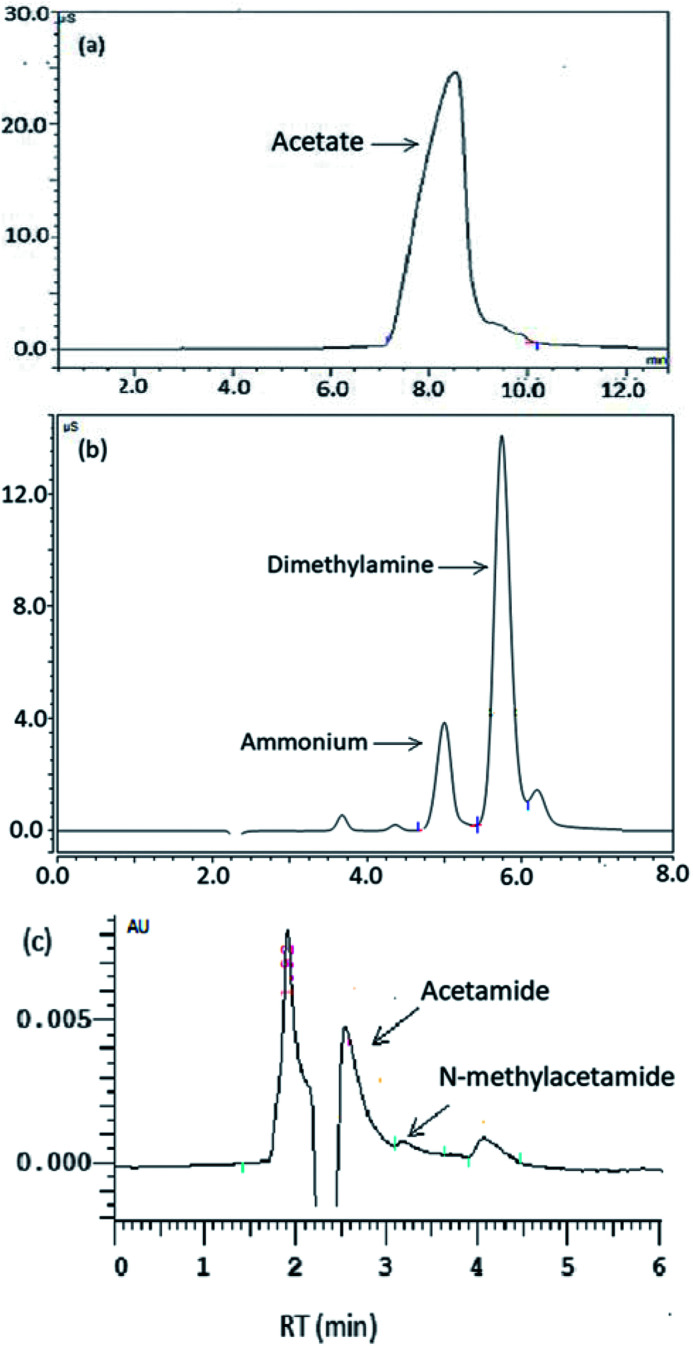
Results showing the anions detected by ion chromatography (a and b) and HPLC (c).

**Fig. 7:**
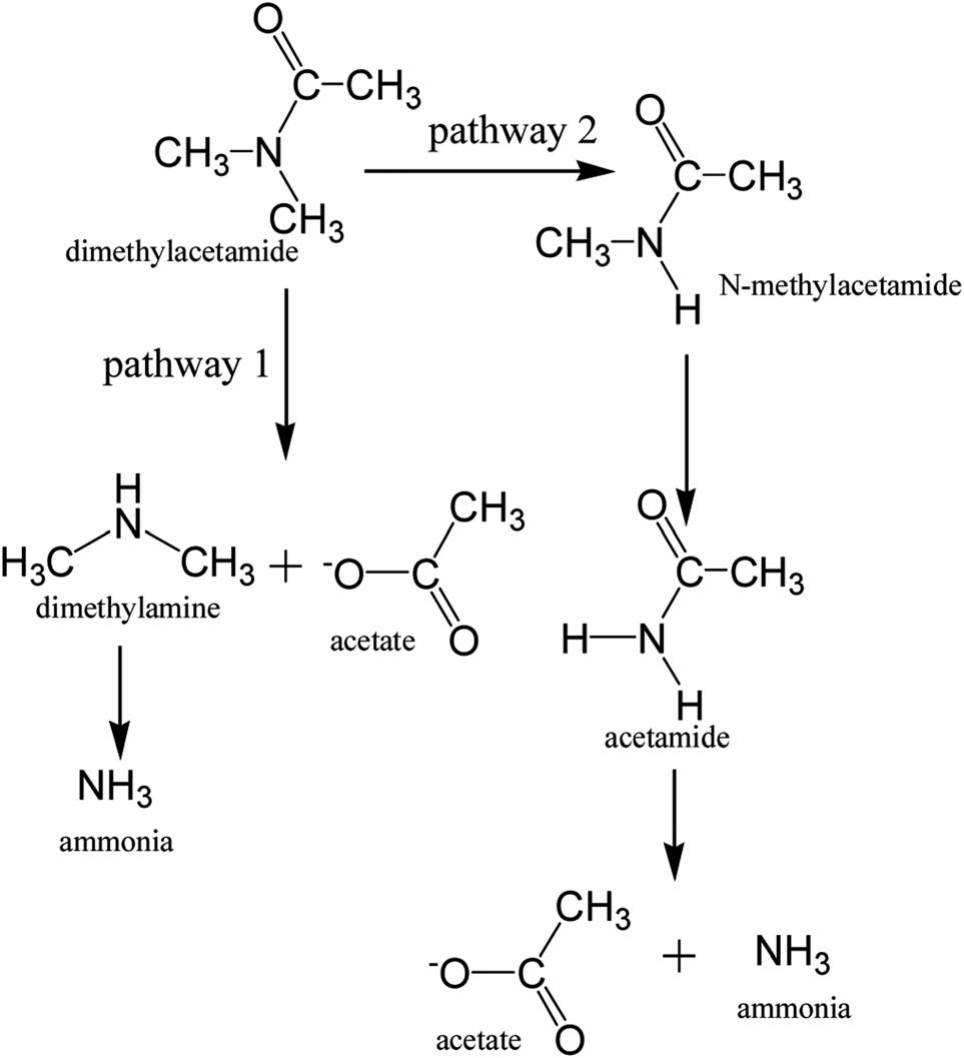
Two proposed pathways for DMAC biodegradation.

DMAC was utilized as a unique carbon and nitrogen source for the growth of microorganisms, supplemented by an external phosphorus source (KH_2_PO_4_). The mechanistic pathway of the DMAC degradation was studied by random analysis of the formed intermediates during the degradation process in the MBR system. Five potential intermediates of DMAC were identified by ion chromatography and HPLC analysis (Fig. 6). Based on the combination of the analysis of the detected intermediates in this study and information in a previous report,^5^ two different degradation pathways of DMAC were proposed and are shown in Fig. 7. All of the intermediates were detected using a reference standard. Moreover, C–N bond cleavage precedes the production of intermediates in both pathways.

**Fig. 8:**
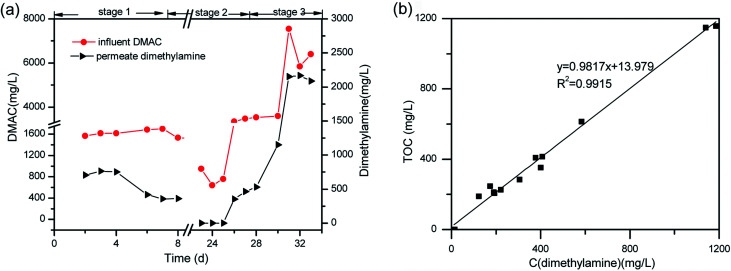
(a) One of the DMAC transformation products (dimethylamine). (b) The linear relationship between the concentration of C_DMA_ and TOC in the permeate.

In order to study the DMAC degradation mechanism in pathway 1, dimethylamine (DMA) and ammonium concentrations were determined using ion chromatography (Agilent 7890A ICS). The accumulation of ammonium (Fig. 2d) and DMA were readily observed at treatment stage 3 (Fig. 8a). While in pathway 2, acetamide and *N*-methylformamide (MMAC) were not readily detectable, probably due to low concentrations. In pathway 1, the degradation of DMA to ammonia^49^ and the conversion of acetamide into ammonia have been previously confirmed.^5,50^ It has also been reported that the maximum accumulation of dimethylamine (DMA) was equal to 62% of the initial dimethylformamide (DMF) concentration.^51^ As can be seen in Fig. 6a, the acetate concentration was too low to accumulate in the A/O-MBR because it is readily biodegradable organic matter.^52^ Nevertheless, during DMAC utilization by the *Rhodococcus* sp. strain B83, the accumulation of acetate ions was detected.^5^ The permeate concentration of C_DMA_ (carbon in DMA) and TOC are directly related, as shown in Fig. 8b. DMA was found to be the main organic component of the effluent. Similarly, accumulation of ammonium in the A/O-MBR treatment system resulted in limited TN removal efficiencies (Fig. 4d). Application of aerobic granular sludge for simultaneous nitritation–denitritation treatment^53^ and anammox processes combined with denitrifying anaerobic methane oxidation (DAMO)^54^ could be considered in order to alleviate the ammonium accumulation in future studies.

## 4. Conclusions

In this study, we showed the feasibility of applying a A/O-MBR system for the treatment of real DMAC wastewater. During the entire operation, 100% DMAC removal could be achieved at a maximum DMAC concentration of 7548 mg L^−1^. COD and TOC removal efficiencies reached 98.4% and 99.3% at influent concentrations of 2011.8 and 717.5 mg L^−1^, respectively. Severe membrane fouling was observed during short-term operation of the A/O-MBR, with protein fractions of EPS being the dominant membrane foulant composition, as confirmed both qualitatively and quantitatively using EEM and protein analysis. Moreover, accumulation of dimethylamine and ammonium was observed in the effluent, which proved that the two DMAC degradation pathways proposed were reasonable. Thus, in the future, integration of a MBR with ammonium-scouring reactors will be highly beneficial to meet discharge requirements in the treatment of DMAC contaminated wastewater.

## Conflicts of interest

There are no conflicts to declare.

## Supplementary Material
